# How should abnormal uterine bleeding be managed in people with bleeding disorders: a systematic review of the literature and thematic synthesis

**DOI:** 10.1016/j.rpth.2025.103167

**Published:** 2025-09-01

**Authors:** Rameen Masood, Vidiya Dev, Millie Gee, Katie Finch, Daniel M. Fletcher, Olufikayo Bamidele, Jo Traunter, David Allsup, Barbara-ann Guinn

**Affiliations:** 1 Hull York Medical School, University of Hull, Hull, United Kingdom; 2 Hull York Medical School, University of York, York, United Kingdom; 3 Centre for Biomedicine, Hull York Medical School, University of Hull, Hull, United Kingdom; 4 Cancer Awareness, Screening and Diagnostic Pathways Research Group, Hull York Medical School, University of Hull, Hull, United Kingdom; 5 School of Education, Faculty of Arts, Culture and Education, University of Hull, Hull, United Kingdom; 6 Hull University Teaching Hospital National Health Service Trust, Queens Centre for Oncology and Haematology, Castle Hill Hospital, Cottingham, United Kingdom

**Keywords:** blood coagulation disorders, hemorrhagic disorders, hemostatic disorders, menstruation disturbances, menorrhagia, systematic review

## Abstract

Abnormal uterine bleeding (AUB) describes any bleeding from the uterus that deviates from the norm in terms of regularity, duration, or volume. AUB is a common condition that can significantly affect quality of life. Although inherited bleeding disorders (IBDs) can cause heavy menstrual bleeding, there is no clear consensus on how AUB is best managed in those patients. This study aimed to address this knowledge gap using evidence based on clinical findings to define the best management of AUB in patients with IBD by conducting a systematic review of the literature. Searches were conducted for articles published from January 1, 2000, until May 6, 2024 in the Embase (PubMed), Medline, Scopus, Cochrane library, Google Scholar, and Cumulative Index to Nursing and Allied Health Literature complete via the Elton B. Stephens Company databases. In total, 244 studies were assessed for eligibility based on inclusion and exclusion criteria. Included studies were appraised for risk of bias and quality assurance using the Newcastle Ottawa Scale, after which data was systematically coded to generate descriptive and analytical themes. Thirteen studies were included in the thematic synthesis, encompassing over 893 participants. Thematic synthesis identified hormonal treatments, such as the levonorgestrel-releasing intrauterine system (LNG-IUS), to be largely effective in the symptom management of AUB in IBDs. Treatment of AUB patients with LNG-IUS, followed by tranexamic acid or 1-deamino-8-d-arginine vasopressin (DDAVP) commonly led to amenorrhea. The use of LNG-IUS as first-line therapy is recommended for those with AUB, followed by the use of combination therapy such as tranexamic acid and desmopressin. We identified the need to strengthen communication between specialists involved in the care of those with AUB and IBDs.

## Essentials

• Patients with bleeding disorders can experience hardship due to AUB.
• A systematic review and thematic synthesis identified best practice managements for patients with AUB.
• Best practice treatment can help improve quality of life for patients with AUB.
• The levonorgestrel-releasing intrauterine system is recommended, followed by combination therapy.

## 1 Introduction

Abnormal uterine bleeding (AUB) refers to any variation from a normal menstrual cycle in terms of frequency, regularity, duration, and volume. AUB can interfere with physical, social, emotional, and material quality of life (QoL) [1]. AUB has been quantified as blood loss of > 80 mL or occurrence of bleeding over a duration of >7 days, but it is widely recognized that a diagnosis of AUB is highly subjective [2]. The International Federation of Gynecology and Obstetrics (FIGO) divided the causes of AUB into structural and nonstructural categories [3]. FIGO noted that the nonstructural cause of AUB was coagulopathy, and notably, inherited bleeding disorders (IBDs). Twenty percent of adolescent people who menstruate present to primary care with AUB will have a bleeding disorder, yet most of these individuals will not undergo testing for an IBD [4].

IBDs are defined as disorders of the blood that cause an increased disposition to bleed; there are many different IBDs of varying severities. The most common IBD is von Willebrand disease (VWD), affecting 1 in 2000 people in the United Kingdom, followed by hemophilia A then hemophilia B [4]. VWD has an estimated prevalence of 16.5 people per 100,000 in the United Kingdom population [5]. AUB can be a symptom of IBD and has been described by many individuals with IBD as having the largest negative effect on their QoL, when considering all their symptoms [6].

Although scientifically there is some awareness of the relationship between AUB and IBD, this does not appear to translate to clinical practice. The European Association for Haemophilia and Allied Disorders state that the underdiagnosis of IBD in people who menstruate is common and that individuals typically experience diagnostic delays of between 8 and 16 years [7]. EAHAD outlines 10 principles that aim to improve health, well-being, and QoL in people with IBD who menstruate. These principles highlight the need for clear pathways from diagnosis to treatment [4].

With regards to managing AUB, the National Institute for Health and Care Excellence (NICE) offers guidance for those without an underlying pathology, such as IBDs, and to those with fibroids [8]. For these patients, NICE recommends the use of hormonal treatment, tranexamic acid (TA), and nonsteroidal antiinflammatory drugs to manage patients with fibroids < 3 cm and with suspected or diagnosed adenomyosis. For patients with AUB with larger fibroids, NICE has more tailored management recommendations [8]. There is an absence of NICE published guidance for the management of AUB in patients with IBDs. Additionally, FIGO have not supported any published recommendations on how to manage AUB in IBD. As discussed, FIGO have only published systems to understand menstrual bleeding to aid clinicians in developing management plans for those with AUB [9].

A UK Haemophilia Centres Doctors’ Organisation published guidelines for the gynecologic management of people with IBD who menstruate in 2022, intended for hematologists and gynecologists involved in the care of patients with AUB and an IBD. They recommend the use of TA in preference to desmopressin to prevent and treat AUB in certain IBDs. Regarding hormonal treatment, the use of the levonorgestrel-releasing intrauterine system (LNG-IUS) is recommended as an effective first-line therapy for AUB in patients with an IBD for those who do not wish to conceive [10].

Not all patients with an IBD require active management with prohemostatic therapies, with such interventions often only required in response to trauma or surgical procedures. The British Society for Haematology recognizes that in some people with VWD, prophylactic therapy is needed to reduce the frequency of bleeding episodes [11]. However, while prophylaxis for bleeding episodes does appear useful in decreasing bleeding events, there appears to be a knowledge gap regarding the efficacy of this therapy on AUB [12].

Therefore, the aim of this study was to use evidence-based clinical findings to define the best management for AUB in individuals with IBDs. To achieve this, a systematic review of the literature was performed.

### 1.1 Objectives

• To systematically review and compare existing clinical guidelines and management strategies for AUB in the context of IBD.
• To emphasize the need for the development of personalized treatment strategies that optimize modality choice based on patient characteristics, choice, and severity of symptoms.
• To identify limitations in current treatment outcome assessments.
• To identify knowledge gaps in current clinical practice and propose evidence-based recommendations for the optimal management of AUB in individuals with IBD.

## 2 Methods

### 2.1 Public and patient involvement

This study was initiated by a single patient with an IBD and experience of AUB. Their priorities, experience, and preferences informed our research question. They were engaged throughout the study and helped inform the inclusion and exclusion criteria. They were given time to engage with the data extraction and read the article as it developed into its final format.

### 2.2 Systematic review

This systematic review adheres to the Preferred Reporting Items for Systematic Reviews and Meta-Analyses (PRISMA) guidelines [13] including the development of a Protocol dataset (Supplementary Information I) and prospective registration with an International Prospective Register of Systematic Reviews (PROSPERO) (CRD42023452533).

#### 2.2.1 Eligibility criteria

The research question was developed using the following PECO framework [14]:

• **P**articipants—adults with AUB and IBD.
• **E**xposure—treatment to manage AUB.
• **C**omparators—patients whose AUB was not managed.
• **O**utcome—effect on QoL of the patient.

Studies assessed were not limited to those in the English language but included participants with experience of AUB and IBD, with no age, geographic location, publication date, or setting restrictions (Table 1). Articles were excluded if AUB was absent and/or bleeding disorders were not diagnosed or had a nongenetic etiology. In addition, reviews and studies of <10 participants/case reports were excluded.

**Table 1:** Exclusion/inclusion criteria.

Inclusion	Exclusion
Primary literature sources, with reviews kept in until screening was completed for the purpose of backward snowballing	Case studies, reviews.
Human studies	Studies on cell lines, animal models of inherited bleeding disorders or heavy menstrual bleeding (HMB).
Patients diagnosed with HMB and a bleeding disorders	Diseases other than HMB.
Patients diagnosed with heavy menstrual bleeding	Diseases other than inherited bleeding disorders.
All ages	
All geographical locations	
All publication dates	
All languages	

Review articles were only removed once the citations in all selected manuscripts had been screened against the inclusion/exclusion criteria. This “backward snowballing” step helped ensure that relevant literature was successfully found as part of a systematic review.

### 2.3 Information sources

We identified articles from 6 online databases (Embase [PubMed], Medline, Scopus, Cochrane, Google Scholar, and Cumulative Index to Nursing and Allied Health Literature) published between 1 January 2000 and 6 May 2024.

### 2.4 Search strategy

The development of the search strategy dataset was based on the index terms found in 3 to 6 sentinel articles retrieved from an initial PubMed screen of the literature. Published manuscripts focusing on AUB and IBD were identified using Medical Subject Headings search terms and variations thereof as follows: ([exp Blood Coagulation Disorders] OR [Clotting disorder∗]) AND ([exp Abnormal Uterine Bleeding] OR [heavy period] OR [Menorrhagia] OR [Heavy menstrual period]). These searches were repeated across all 6 databases.

### 2.5 Selection process

Article selection was performed by 2 independent pairs of screeners; group 1: R.M. and V.D. until September 2023, and group 2: M.G. and K.F. from September 2023 to May 2024. Both pairs of screeners discussed any queries regarding differences in selected publications. Excel was used as a repository for articles, and after deduplication, the articles were screened by title, abstract, and full text against the inclusion and exclusion criteria (Table 1).

### 2.6 Risk of bias assessment

The Newcastle Ottawa Scale (NOS) for Assessing the Quality of Nonrandomized Studies in Meta-Analysis was used to perform quality assurance assessment of the selected studies [15]. The studies were assessed based on selection, comparability, and outcome and were ranked from 0 to 4 stars (indicated by ∗). Zero stars signifies a lack of information required, and 4 stars signifies a perfect match to the criteria, with no indication of what could be improved upon.

### 2.7 Data extraction

For standardization, a data extraction form was piloted on Excel using several selected studies with input from all reviewers. This included fields for study methodology, interventions/managements being reviewed, sample size, and qualitative experience. The pairs of screeners performed data extraction on the articles they selected and agreed upon.

### 2.8 Thematic synthesis

Based on previous guidance [16], we performed the thematic synthesis of data in 3 stages. Data from each article was coded and reviewed to identify key concepts, using text directly from the selected articles. Concepts included the management options and recommendations regarding best practice in the management of AUB and IBD, as presented by each article. Similar concepts within and between articles were grouped to create descriptive themes. Reviewers weighted themes according to the frequency in which they appeared across the articles. The greater number of times a theme was identified, the more weight was given to it. Comparisons between themes were made, and these were subjectively interpreted in accordance with our research intentions to synthesize analytical themes. This was conducted collaboratively by 3 reviewers (V.D. R.M., and M.G.) using the Excel data extraction form.

### 2.9 Ethics

To ensure we met recognized standards of ethical considerations, only articles that had followed their own country’s ethical standards were included in this research.

## 3 Results

### 3.1 Screened studies selected for systematic review

The search amassed 11,568 papers, of which 5671 were duplicates (Figure). We used prespecified inclusion/exclusion criteria (Table 1) to screen articles based on title and abstract, resulting in 244 articles being selected. At the stage of abstract reading, our search excluded books, systematic reviews, meta-analyses, and conference papers. The articles were then screened by full text, with 16 meeting the eligibility criteria to be included in further analysis (Supplementary Information II). During full-text analysis, articles with only abstracts and those that did not meet the inclusion/exclusion criteria were removed.

**Figure:**
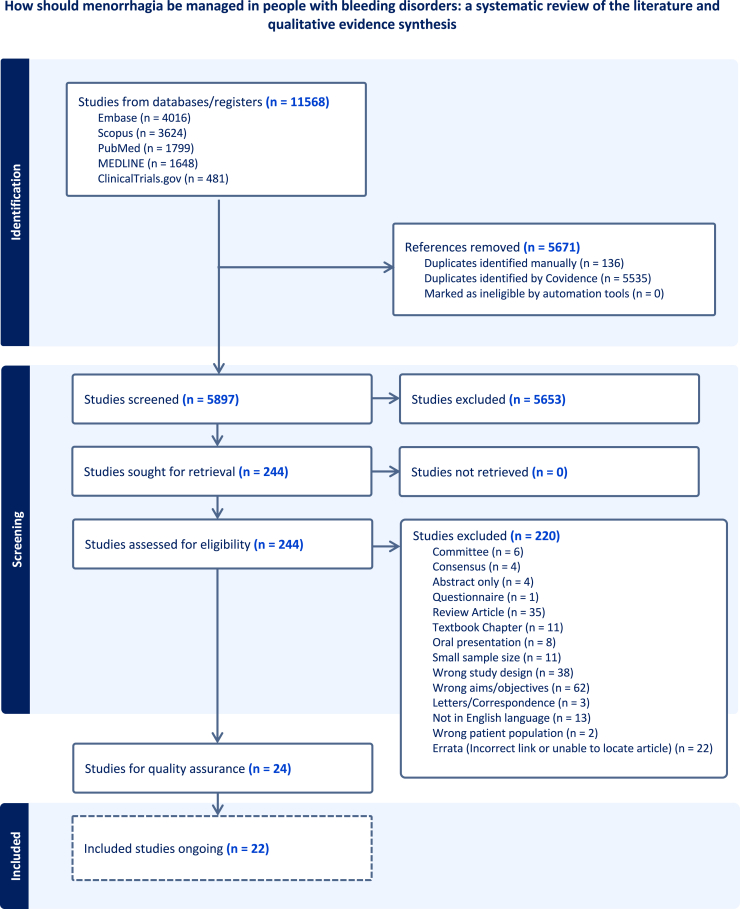
Preferred Reporting Items for Systematic Reviews and Meta-Analyses (PRISMA)-P flow diagram. Flowchart of the screening process for articles that had considered the management of people with heavy menstrual bleeding and bleeding disorders.

Reviews remained in the systematic review until it was completed, these were then used for a backward snowballing step [17] (Supplementary Information III), and we found 35 articles that had been excluded during our original searches, which were then fully assessed. Any disparities in chosen articles were discussed between the reviewers and a consensus achieved. However, when disagreements remained, they were resolved by a third reviewer (B.G.). Articles that could not be accessed were sought from the corresponding author and, where different, the senior author.

### 3.2 Quality assurance

The full-text versions of 16 studies on AUB and IBD were assessed for eligibility and quality using a modified NOS template (Table 2) [[18], [19], [20], [21], [22], [23], [24], [25], [26], [27], [28], [29], [30]]. Three of them were excluded considering the risk of bias and applicability (Table 3) [18,19,21], and all others met the requirements for data extraction (Supplementary Information IV). This identified 13 studies that met the quality assurance criteria for data extraction.

**Table 2:** Selection, comparability, and outcome assessment applied to the selected studies.

Reference	Selection (/4)	Comparability (/2)	Outcomes/Exposure (/3)	Rating
Campos et al., 2020 [29]	∗∗∗	∗	∗∗∗	Good
Chi et al., 2011 [26]	∗∗	∗	∗∗	Fair
De Wee et al., 2011 [18]	∗∗	∗∗	−	Poor
Dietrich et al., 2017 [20]	∗∗	∗	∗∗	Fair
Dowlut-McElroy et al., 2015 [21]	∗∗∗	∗	∗	Poor
Govorov et al., 2016 [30]	∗∗∗	∗	∗∗	Good
Greenmyer et al., 2021 [19]	∗∗	∗	−	Poor
Huguelet et al., 2022 [22]	∗∗∗	∗	∗∗	Good
Huq et al., 2012 [27]	∗∗∗	∗∗	∗∗	Good
Kadir et al., 2002 [28]	∗∗∗	∗	∗∗	Good
Leissinger et al., 2001 [23]	∗∗∗	∗∗	∗∗	Good
Pennesi et al., 2020 [24]	∗∗∗	∗∗	∗∗	Good
Ragni et al., 2023 [25]	∗∗∗	∗∗	∗∗	Good

Newcastle-Ottawa Scale (NOS) guidelines for cohort studies were followed. Studies with 0 stars were subsequently excluded from further meta-analysis. Thresholds for converting the NOS to Agency for Healthcare Research and Quality standards (good, fair, and poor):.

*Good quality*: 3 or 4 stars (∗) in selection domain AND 1 or 2 stars in comparability domain AND 2 or 3 stars in outcome/exposure domain.

*Fair quality*: 2 stars in selection domain AND 1 or 2 stars in comparability domain AND 2 or 3 stars in outcome/exposure domain.

*Poor quality*: 0 or 1 star in selection domain OR 0 stars in comparability domain OR 0 or 1 stars in outcome/exposure domain.

/3 refers to the score for that criteria being out of a possible 3 stars, /2 refer to the score for that criteria being out of a possible 3 stars, /4 refers to the score for that criteria being out of a possible 4 stars.

**Table 3:** Articles removed during quality assurance and risk of bias analysis.

Reference	Reason for exclusion—poor rating
De Wee et al., 2011 [18]	This was a cross-sectional study and bleeding severity was measured using a given score.
Dowlut-McElroy et al., 2015 [21]	No real evidence of follow-up with the cohort.
Greenmyer et al., 2021 [19]	Could possibly touch upon this new type of management, which can be considered for treating heavy menstrual bleeding in patients with bleeding disorders.

### 3.3 Data extraction

A total of 1069 participants were included in the 13 studies. The largest study involved 423 participants in the Netherlands, and the smallest study had 11 participants and was conducted in the United States [18,19]. Seven studies were undertaken in the United States [[19], [20], [21], [22], [23], [24], [25]], 3 in the United Kingdom [[26], [27], [28]], 1 in Brazil [29], 1 in the Netherlands [18], and 1 in Sweden [30]. There were no studies that only examined qualitative data; however, 6 studies performed quantitative analysis only [18,19,21,22,24,29] (Supplementary Information IV), and 7 studies conducted both qualitative and quantitative analyses [20,[25], [26], [27], [28],^30^]. The qualitative analysis focused on discussing lived experiences of patients, and the quantitative analyses primarily determined scores for the Pictorial Blood Assessment Chart (PBAC) [[25], [26], [27], [28], [29], [30]], blood tests [27,29], QoL [18,23,[25], [26], [27], [28], [29], [30]], and/or other measures [19,21,22,24] (Supplementary Information VA).

### 3.4 Thematic synthesis

Patients analyzed in these clinical settings expressed a range of emotions concerning the management of AUB in the context of their bleeding disorders. Four key analytic themes were identified reflecting patients’ experiences with AUB and bleeding disorders during diagnosis, treatment and symptom management (Supplementary Information VB and VC).

#### 3.4.1 Restoring control

Tackling AUB. For people with IBD who menstruate, AUB is more than just a clinical issue and in many cases is a persistent disruption to daily life. The advent of the LNG-IUS has provided many people who menstruate with renewed hope, enabling them to regulate their cycles, significantly mitigating blood loss and enhancing overall QoL. Five studies [18,19,26,29,30] within this systematic review affirmed that LNG-IUS is highly effective in managing AUB.

Campos et al. [29] reported that “The use of LNG 52-mg IUS was effective in reducing menstrual bleeding in the people who menstruate with IBD by 3- months from baseline” and further mentioned “The median PBAC score was higher before LNG 52-mg IUS placement than at 3, 6, and 12 months after placement (*P* < .001), demonstrating its efficacy.”

Chi et al. [26] addressed the practicalities of introducing the LNG-IUS as a management option for individuals with AUB and IBDs, stating that the LNG-IUS “can be easily inserted in a clinic setting and is well accepted by people who menstruate. It should be offered to people who menstruate with IBDs who also require contraception and certainly prior to surgical options.”

#### 3.4.2 Nonhormonal treatment and other therapies

Nonhormonal therapies provide an important alternative for managing AUB in patients with IBD, particularly for those who cannot tolerate or prefer to avoid hormonal treatments. Ten studies [18,19,21,[23], [24], [25], [26], [27], [28], [29]] explored a wide range of nonhormonal and alternative treatment strategies, including desmopressin, TA, and iron supplementation and offered insights into their mechanisms, efficacy, and role in personalized patient care.

Combination therapy is an alternative that can be considered for managing AUB, benefiting patients by integrating both hormonal and nonhormonal agents to achieve efficient symptom control. Dowlut-McElroy et al. [21] recommended “that strong consideration be given to combination non-hormonal and hormonal modalities for the treatment of HMB (heavy menstrual bleeding) in people who menstruate with bleeding disorders.”

For people who menstruate who do not wish to preserve their fertility, endometrial ablation can be considered. Huq et al. [27] concluded that “Endometrial ablation appears to be a safe and effective long-term treatment for HMB in people who menstruate with IBDs. It significantly decreases menstrual blood loss and improves QoL.”

DDAVP (1-deamino-8-d-arginine vasopressin) was explored as an option to manage and prevent AUB in IBDs. Leissinger et al. [23] suggested that “when used for the treatment of menorrhagia, the efficacy of high-dose DDAVP intranasal spray (1.5 mg/mL) was rated as “excellent” after 655 (92%) of 721 daily uses.” However, Kadir et al. [28] also explored the use of the DDAVP nasal spray but found no statistically significant improvement in the management of AUB in IBDs.

Recombinant von Willebrand Factor (rVWF) is another nonhormonal treatment option worthy of consideration. Ragni et al. [25] compared the effectiveness of rVWF and TA for the management of AUB and found that “rVWF is inferior to TA in reducing HMB in subjects with mild or moderate VWD, with neither treatment showed a clinically relevant effect.” This finding emphasizes the limited evidence base surrounding rVWF as a prospective treatment for AUB. Given the potential contraindications to hormonal therapy in individuals with IBDs, particularly those with thrombotic risk factors, the need for alternative therapies such as rVWF is an interesting and rather critical avenue for further research. Future studies should explore whether rVWF, possibly in combination with other agents, could offer a more tailored and safer alternative for AUB management.

#### 3.4.3 Reclaiming QoL

How to shift from struggle to strength. AUB not only causes physical distress but also profoundly impacts various facets of an individual’s life, including career choices, mental health, and social participation. The studies analyzed in this systematic review demonstrate that LNG-IUS significantly improves QoL, with notable improvements in physical functioning, emotional well-being, and pain reduction. In addition, patients with AUB are at an increased risk of developing iron deficiency anemia, a chronic condition that can profoundly impact their QoL. The resulting fatigue and, in some cases, cognitive impairment, can further exacerbate daily challenges, limiting their ability to perform routine activities and affecting overall well-being. Therefore, the ability to resume activities of daily living without constant worry about heavy bleeding represents a transformative change for many patients.

Eight studies [18,[24], [25], [26], [27], [28], [29], [30]] utilized the Rand 36-item short form survey, which scores 8 health concepts: physical function, bodily pain, role limitations due to physical health problems as well as personal or emotional problems, emotional well-being, social functioning, energy/fatigue, and general health perceptions as well perceived change in health. QoL was found to be improved through the insertion of LNG-IUS as follows, “There was an improvement in all 8 parameters of QoL (*P* < .001). The mean hemoglobin, ferritin, and serum iron levels were also higher at 12 months than before LNG 52-mg IUS placement” [29]. These results indicate that patients with AUB may commonly have iron deficiency without anemia, as proposed by others [31]. Huq et al. [27] highlighted the improvement in QoL after endometrial ablation: “The overall scores for all categories included in the QoL improved significantly after treatment (*P* < .01). Prior to ablation, 67% rated their general health as poor compared with 0% at follow-up.”

Pennesi et al. [24] stated that “Young people who menstruate with HMB miss more days of school and have increased disruption of hobbies and activities and decreased sports participation because of menses, compared with their peers with normal menses.” This typifies how AUB negatively impacts the lives of individuals with IBD and that managing AUB in those with IBD contributes to an improvement in their QoL.

#### 3.4.4 Rethinking menstrual health

A call for better awareness and research. Gynecological health, particularly in the field of menstrual conditions and IBD, is an underfunded and underresearched area. Seven studies [18,20,21,24,25,27,30] highlighted the importance of increased education and awareness. A range of management options should be considered and the most efficacious chosen based on individual symptoms and underlying pathology.

One of the strategies being assessed is the use of an iPod Touch (Apple Inc) device (ITD) to monitor treatment compliance levels. ITD is predicted to help “improve educational access through engaging technology” [20]. ITD can be introduced and disseminated at various centers and is a useful tool for “managing adolescents with HMB and bleeding disorders by virtue of its inherent adaptability, minimal need for training, low cost, and potential to improve patient compliance with treatment regimens” [20].

Good communication between clinicians involved in the care of patients with AUB and IBD was highlighted, as “close liaison with the hemophilia team is essential to determine the risk of bleeding and the need for hemostatic prophylaxis” [27]. The article described the need for management to include a discussion between hematology and gynecology or the general practitioner to provide best care. It was proposed that communication needs could also be met by “the creation of a national registry that standardizes the collection of menstrual data and treatment plans would further delineate menstrual patterns and effective treatment of HMB in people who menstruate with bleeding disorders” [21].

Counseling patients of their treatment options was a common recommendation, noted particularly in 3 articles [21,24,30]. A significant number of individuals with AUB and IBDs appear not to be involved in the discussion surrounding their own management options, when a plan was being developed. Of note, “Only 28% of the cohort had notations indicating that a treatment plan for HMB had been discussed with their provider prior to menarche (*P* = .0001)” [21].

## 4 Discussion

To address our research question, we performed a systematic literature review of the management of AUB in patients with IBDs that identified 13 full text articles as meeting our criteria. We found 4 key themes among these articles, highlighting various treatment modalities and their effectiveness.

LNG-IUS [18,19,26,29,30] was the most common treatment given to patients, which highlights its importance as a long-term approach due to its dual benefits of reducing bleeding and potentially decreasing the requirement for repeated and more invasive interventions. Additionally, these articles [18,19,26,29,30] emphasized that LNG-IUS offered a better patient experience as it enabled greater autonomy and convenience due to its minimal requirement for maintenance. Patients appreciated that the IUS could remain in place for many years, particularly beneficial for patients who required the continuous management of AUB.

One additional point of interest relates to the expulsion of the intrauterine device before the completion of the treatment period, highlighted in the literature. It is worth considering that this could potentially introduce bias into treatment outcome assessments, as early removal of the intrauterine device may have resulted in incomplete treatment or inadequate follow-up data, impacting the overall effectiveness and comparison of treatments. Gynecologic guidelines need to be updated, and it is essential to critically consider whether long-term oral medications are leading to beneficial outcomes or whether it would be more advantageous to introduce LNG-IUS intervention at an earlier stage. In addition, it is crucial to improve access to this treatment option, especially for those who menstruate who have historically been dismissed or left with few viable solutions.

Leissinger et al. [23] examined the subjective patient experience with vasopressin/desmopressin nasal spray, focusing on patient-reported satisfaction rather than providing quantitative clinical outcomes. The absence of objective treatment outcomes makes it difficult to draw definitive conclusions regarding the clinical efficacy of these treatments. The efficacy of TA and desmopressin nasal spray seemed to be associated with positive outcomes for individuals with AUB and an IBD [21,26]. TA, an antifibrinolytic, is widely used for its affordability and minimal side effects. One of the studies [23] compared rVWF and TA in a randomized crossover trial. The study found that while both treatments reduced PBAC scores and the frequency of flooding, rVWF achieved a slightly smaller reduction in AUB by absolute PBAC score compared with TA. Although neither treatment was able to completely normalize PBAC scores, both made significant improvements in symptom severity. Importantly, QoL and health care utilization were comparable between the 2 groups. Given its cost-effectiveness and comparable efficacy, TA emerged as a more practical option for AUB management, particularly for patients looking for a balance between treatment impact and affordability. Endometrial ablation and octreotide treatment [19,27] were also demonstrated to have varying degrees of success in addressing AUB in patients with VWD. These studies did not include direct comparisons with other therapies and therefore lacked the comparative data necessary for a broader analysis of their relative efficacy.

More innovative approaches to management and to monitor compliance were also considered, including the use of ITD to track and manage AUB in patients with IBDs [20]. Participants utilized the ITD to record information about their IBD based on what they learned from IBD-related websites, as well as track their menstrual cycles and medication usage. This electronic data, coupled with charts of records, provided a closer insight into compliance with prescribed treatment regimens. According to the study, this self-monitoring approach guided and supported the patients, helping them to remain proactive in managing their symptoms, promoting better adherence to treatment, and allowing adjustments based on real-time data. Nonetheless, the limitation with this study was that it did not report relevant treatment outcomes. Although the outcomes from this study are important, they do not directly address the effectiveness of specific treatments in managing AUB, thus excluding the study from a comparative analysis of treatment efficacy.

Three studies [22,26,29] provided data on the proportion of patients achieving amenorrhea after treatment. Campos et al. [29] and Chi et al. [26] did not quantify the amount of bleeding before and after treatment, whereas Huguelet et al. [22] provided an in-depth report on the rate of amenorrhea among young adults and concluded that in individuals with high rates of amenorrhea, LNG-IUS is an effective treatment consideration. Pennesi et al. [24] failed to provide specific data, and the study was ultimately deemed unusable for inclusion in the meta-analysis. Furthermore, the study by Huguelet et al. [22] was paywalled, and despite efforts to access the data, we were unable to obtain the necessary information for review, which further limited the available evidence.

Currently, there are no readily available guidance regarding the optimal management of individuals with IBD that present with AUB. The NICE guidelines [8], which are widely used by gynecologists for managing AUB, offer only 2 nonhormonal treatments, namely TA and nonsteroidal anti-inflammatory drugs. Hormonal treatments include combined hormonal contraception and cyclical oral progestogens. Surgical options include second-generation endometrial ablation and hysterectomy. Moreover, current guidance only supports the investigation of coagulation disorders in those who present with AUB without a current diagnosis of an IBD.

One of the studies [21] compared the efficacy of hormonal treatment with nonhormonal treatment, elucidating that, while hormonal therapy was used as the initial treatment method for just over half the affected population, combination approaches were favored for ongoing treatment.

To our knowledge, this is the first review to explore the management of AUB in individuals with IBDs. Based on the data generated, guidance should be put into place whereby the Royal College of Obstetricians and Gynaecologists can act accordingly when encountering a patient with an IBD experiencing AUB, to manage the symptoms, improve patient outcomes, and improve overall QoL. Ultimately, prior to considering any treatment, it is pertinent to address patients’ concerns about AUB and bleeding disorders. Empowering patients so that they can actively manage their symptoms and find ways to tackle any unexplained symptoms helps, and the World Federation of Hemophilia [32] and UK Haemophilia Centres Doctors’ Organisation [10] are a source of guidance on this for patients and doctors.

As forementioned, FIGO note a nonstructural cause of AUB is an IBD. However, in the NICE guidelines [8], the cause of AUB is not discussed; it is only mentioned to investigate for coagulation disorders if someone presents with AUB to rule it in or out as a cause. There is no guidance on how to treat and manage someone with an IBD who presents with AUB, leaving a knowledge gap that can severely impact clinical practice and thus lead to negative or unwanted patient outcomes. In addition to this, the Royal College of Obstetricians and Gynaecologists [33] does not address the management of AUB in people with IBD who menstruate. Despite this, the Haemophilia Society recognizes that AUB is a common debilitating symptom of someone with a coagulation disorder and acknowledges that this can severely impact QoL. The Haemophilia Society set up a “Talking Red” campaign [4] urging people to come together and talk about bleeding in people who menstruate, to allow them to receive the treatment they require.

Therefore, it is essential for future guidelines in this field to integrate menstrual management into workplace and school health programs. Recognizing LNG-IUS within public health policies can serve as a key tool for improving the overall well-being of people who menstruate. It is crucial to balance effective treatment against side effects, to achieve a meaningful amelioration in the well-being of patients.

### 4.1 Limitations

Although comprehensive, this review had limitations. Certain articles were excluded by virtue of the exclusion criteria, including the screening out of case reports, reviews, and abstracts from conferences. This may have limited the generalizability of our results.

The inclusion of patients with IBD in some studies was not clear prior to reading the full article, and thus these articles were excluded prior to this step [34]. To overcome this, we performed a final search on treatments and IBD and AUB, as well as a backward snowballing step, and found 35 articles that had been excluded during our original searches, which were then fully assessed.

We started our literature search from 1 January 2000 ensuring we encompassed almost 24 years of clinical experience of treating AUB in patients with IBDs. We could have searched further back in time but questioned the relevance of such articles to current practices. We excluded studies involving pediatric populations [35,36], as their physiological parameters differ significantly from those of adults, which could have introduced additional confounding factors. However, menstruation often starts in childhood and would be first treated then. Indeed, some articles did not define how many patients were in each age group except that they included patients aged <21 years.

While platelet function disorders can be both inherited and acquired, certain studies grouped all platelet function disorders together. This may have introduced variability in the included populations, potentially impacting the generalizability of our findings.

Additionally, although our MeSH terms were very comprehensive, there may be some obscurity on the definition of “abnormal uterine bleeding” as our primary focus was AUB rather than abnormal bleeding. This could have restricted the results extracted from articles on clinical practice, as the treatment strategies for AUB can differ depending on the underlying etiology.

Another limitation was the lack of detailed patient outcome data in many studies, limiting our ability to extract outcome data from articles. Without access to raw data, we were unable to analyze all potential outcomes comprehensively, leading to gaps in our quantitative synthesis. In addition, the limited number of available studies and the scarcity of quantitative data constrained our ability to conduct statistical comparisons.

Finally, we excluded certain articles [37,38] because they were reviews rather than primary research studies. Our focus was on data from original experimental or clinical research to ensure the validity of any meta-analysis.

We recognize that the use of IBD as a key criterion in our review does present challenges. IBDs are not a single disease, and there is heterogenicity between the types and severities of IBDs. This may have impacted the validity of the study as the different types and severities of IBD may have varying impacts on HMB and its response to treatment. However, we chose to address IBDs as one entity because many IBDs are rare and difficult to diagnose, meaning sample sizes from individual studies would have been too small or diagnoses too ambiguous to draw useful data from.

Despite these limitations, our study provides valuable insights into the treatment modalities for adolescents presenting with AUB. Future research with standardized definitions, detailed patient outcome reporting, and access to raw data would enhance the robustness of systematic reviews in this field.

## Conclusion

Addressing the knowledge gaps in the management of AUB, particularly in individuals with IBD, is essential for improving physical, mental, and social well-being. While there are recommendations for people with AUB, guidance for those with IBD remains limited. Below, we outline 5 key suggestions, informed via our research, with proposed strategies for implementation in the clinical setting.

### 5.1 LNG-IUS as first-line therapy

LNG-IUS is a highly effective treatment for AUB and should be considered as first-line treatment for individuals with IBD, given its ability to significantly reduce menstrual blood loss while providing long-term symptom control. While every patient should be assessed individually based on their symptoms to determine their suitability for LNG-IUS, there is growing evidence that introducing this therapy earlier in the treatment pathway, rather than as a last resort after other therapies have failed, leads to better symptom control and improved QoL. Delaying its use until symptoms have significantly worsened may result in unnecessary prolonged distress for patients.

To implement this, gynecologists should proactively discuss the LNG-IUS as an early treatment option, with training programs emphasizing shared decision making to ensure patients understand the benefits and potential side effects of early LNG-IUS insertion. Additionally, improved access to specialist consultations in primary care settings would facilitate timely referrals and earlier intervention. LNG-IUS can be used as a contraceptive for up to 8 years depending on brand. Basic counseling should be provided about the expected bleeding pattern after insertion. Irregular bleeding may occur for up to 6 months after insertion and often decreases to 1 light day of bleeding each month within 1 year.

### 5.2 The use of combination therapy

For patients in whom LNG-IUS is not suitable or insufficient, combination therapy with TA, desmopressin, or other agents should be explored. However, clinicians must remain cautious of venous thromboembolism risks associated with certain treatments. To implement this, a clear, evidence-based treatment algorithm should be developed, outlining when and how combination therapies should be used. It is pertinent to also incorporate risk stratification protocols into the clinical guidelines to ensure a considered balance between efficacy with safety.

### 5.3 Strengthening counseling and communication between specialists

Managing AUB in individuals with IBDs requires more than just prescribing the right treatment. Open, effective communication between specialists and a patient-centered approach to care is equally vital. Too often, patients find themselves caught between different specialties, with gynecologists focused on managing menstrual symptoms and hematologists prioritizing bleeding risks. A lack of coordination can lead to delays in treatment, conflicting advice, or even overlooked concerns that significantly impact a patient’s well-being.

To provide truly individualized care, discussions about treatment options should happen early, and counseling should be incorporated to ensure patients feel heard, understood, and involved in their own care. To successfully implement this, a centralized communication system can be developed that would help streamline care and ensure specialists are continually updated about a patient’s health. This could be further supported by clear national guidelines that encourage collaboration between gynecologists, hematologists, and general practitioners and could be facilitated by joint clinics. More practically, simple steps such as ensuring all patients have a named point of contact within each specialty and access to written treatment plans could make a tremendous difference in their confidence in managing their condition.

### 5.4 Raising awareness through public and patient education

Education is key. Due to the lack of sufficient public awareness, many individuals with AUB delay seeking medical advice, assuming their symptoms are “just normal” or something they must endure. This is even more pronounced for those with IBDs, for whom a delayed diagnosis could potentially have serious long-term consequences. To combat this, a multifaceted approach needs to be designed.

As part of this approach, it will also be valuable to assess patients’ treatment compliance. As highlighted in the study by Dietrich et al. [20], iPods or similar technologies could be implemented to educate patients about their condition. Additionally, these devices can facilitate real-time data collection, enabling specialists to monitor patients’ health more effectively and promptly identify any untoward activity.

Moreover, clinics and hospitals could introduce more patient-friendly materials such as leaflets, digital resources, and visual guides targeted to those experiencing symptoms of AUB. Social media and community outreach programs could also play a contributory role, particularly in reaching younger individuals who may not otherwise seek help. Collaboration with schools and primary care settings could further increase awareness, ensuring that conversations about menstrual health are normalized rather than stigmatized. It is indispensable to acknowledge that while these initiatives are crucial, we currently do not have concrete evidence proving that awareness campaigns directly lead to better outcomes. Nevertheless, equipping people with the knowledge to recognize their symptoms and seek timely care has the potential to make a profound difference, both in improving individual outcomes as well as reducing the burden of delayed diagnoses.

### 5.5 Need for further research and discussion

Despite existing treatments, gaps remain in understanding the broader implications of AUB management in individuals with IBDs. More research is needed on long-term treatment outcomes, side effects, the mental health impact of different therapies, and the logistical challenges of implementing new guidelines in the clinical settings. Future studies should prioritize patient-centered research, including longitudinal studies assessing treatment compliance, satisfaction, and QoL outcomes. Research should also continuously explore innovative therapies and nonhormonal alternatives to provide more tailored treatment options. Overall, there is a critical need for more focused research to optimize treatment options, promote early diagnosis, and improve overall management strategies.

AUB is both a symptom and condition within itself that should be better understood in theory through regular review so that its management can be better explored in clinical practice. Based on the data extracted, guidance should be put into place whereby primary care doctors follow NICE guidelines [8] when encountering a patient with an IBD experiencing AUB. Our recommendations are based on current guidelines used by gynecologists, which should be shared with hematologists treating patients with IBDs.

## Supplementary material

Supplemental Protocol
