# Supplementary material for: How should abnormal uterine bleeding be managed in people with bleeding disorders: a systematic review of the literature and thematic synthesis
Source: Res Pract Thromb Haemost. 2025 Sep 1;9(6):103167. doi: 10.1016/j.rpth.2025.103167 (PMC12495142; doi:10.1016/j.rpth.2025.103167)
Supplement: Supplemental Protocol [file mmc1.docx]

SLR Protocol

**Title:**

How should menorrhagia be managed in women with inherited bleeding disorders: a patient questionnaire, systematic review and meta-analysis.

**Registration:**

This systematic literature review is registered by the registry PROSPERO. The registration number is: [CRD42023452533](C:\\Users\\535212\\AppData\\Local\\Microsoft\\Windows\\INetCache\\Content.Outlook\\VQSE6W62\\crd.york.ac.uk\\PROSPERO\\display_record.php?RecordID=452533)

**Authors:**

Dr Barbara Guinn. Centre for Biomedicine, The University of Hull. Email: [Barbara.Guinn@hyms.ac.uk](mailto:Barbara.Guinn@hyms.ac.uk). Address: Centre for Biomedicine, Hull York Medical School, University of Hull, Hull, HU6 7RX.

Dr Jo Traunter. The University of Hull. Email: [J.Traunter@hull.ac.uk](mailto:J.Traunter@hull.ac.uk). Address: Hull York Medical School, University of Hull, Hull, HU6 7RX.

Dr David Allsup. Hull York Medical School. Email: [David.Allsup@hyms.ac.uk](mailto:David.Allsup@hyms.ac.uk). Address: Hull York Medical School, University of Hull, Hull, HU6 7RX.

Vidiya Dev. Hull York Medical School. Email: [hyvd2@hyms.ac.uk](mailto:hyvd2@hyms.ac.uk). Address: Hull York Medical School, University of York, Heslington, York, YO10 5DD.

Rameen Masood. Hull York Medical School. Address: Adress: Hull York Medical School, University of York, Heslington, York, YO10 5DD. Hull York Medical School, University of York, Heslington, York, YO10 5DD.

Millie Gee. Hull York Medical School. Email: [hymg24@hyms.ac.uk](mailto:hymg24@hyms.ac.uk). Address: Hull York Medical School, University of York, Heslington, York, YO10 5DD.

Katie Finch. Hull York Medical School. Email: [hykf7@hyms.ac.uk](mailto:hykf7@hyms.ac.uk). Address: Hull York Medical School, University of York, Heslington, York, YO10 5DD.

The guarantor of review will be Dr Barbara Guinn.

All authors will contribute to oversight of this review.

**Support:**

Sources of financial support include the INSPIRE programme, managed by the Academy of Medical Sciences and funded by the Wellcome Trust. These do not have any involvement in the development of this protocol.

**Introduction:**

Menorrhagia is a medical term that refers to prolonged or excessive menstrual bleeding. To categorise abnormal blood flow, one must initially define what constitutes normal. Generally, menstrual bleeding lasts for 3-7 days, but in women suffering from menorrhagia, the bleeding lasts longer than seven days, and women typically lose more than 80mL of blood per menstrual cycle. Menorrhagia has been strongly associated with deteriorating health and adverse effects on daily routine in women suffering from a bleeding disorder. Around 7000 women in the UK are suffering from a inherited bleeding disorder known as von Willebrand disorder (VWD). This is a haematological disorder distinguished by impaired blood clotting. Menorrhagia and bleeding disorders, most commonly von Willebrand disease, affect many women around the world and so it is significant to receive a deeper appreciation into the severity of the problem as well as women's experiences.

We would like to uncover to what extent the impact of heavy menstrual bleeding has on the lives of biological females with bleeding disorders to help inform how menorrhagia management in women with bleeding disorders could be improved.

The primary outcome of this systematic review is to explore and understand how menorrhagia is being managed in women with bleeding disorders, and how it could be improved in the future. The measure of effect will be the frequency of heavy menstrual bleeding in patients with inherited bleeding disorders. Qualitative analysis of current best practice and lived experiences from patients. An additional outcome includes possible recommendations for future treatment and healthcare engagement. The measure of effect for this outcome will be the frequency of heavy menstrual bleeding in patient groups, risk difference, and number needed to treat. We will consider the financial cost to the healthcare system.

**Methods:**

Studies using qualitative and quantitative data will be analysed to ensure varied and rigorous results. This strategy will further provide an exhaustive comprehension and insight into the topic. The design of the studies will also be considered whether they are observational studies, randomised controlled trials (RCTs), cohort studies or cross-sectional studies. According to the hierarchy of evidence pyramid, systematic reviews and RCTs hold the most reliability and negligible bias; therefore, such studies will be primarily preferred and prioritised.

The participants and population of this study are to include girls and women diagnosed with bleeding disorders such as von Willebrand disease (VWD), haemophilia and inherited platelet disorders including MYH-9 disorders, Glanzman's thrombasthenia, Bernard Soulier and inherited macrothrombocytopaenia. From this study we will exclude:- Women with acquired disorders such as immune thrombocytopenia (ITP) will be excluded and those women who have an iatrogenic cause such as concomitant treatment with an anticoagulant or anti-platelet agent. Additionally, women over the age of 50 (post-menopause) and studies with a sole focus on pregnant women (pregnancy can impact bleeding patterns) will also be excluded.

A variety of studies will be included, especially those aimed at exploring interventions for the management of menorrhagia in women with bleeding disorders. Inclusion criteria includes those who are on treatment or have been referred to tertiary care. There will be no comparator or control groups.

Two medical students (MG and KF) will independently work on examining various information sources and then determining the suitability of identified studies. Results will be matched against the eligibility criteria to analyse their suitability. The medical students will use multiple online databases to perform an extensive literature search, such as PubMed, Scopus and OVID. Titles and abstracts will be analysed to assess their relevance to the research question (articles not eligible will be excluded).

In addition, to consider a study entirely relevant, it will include terms relating to menorrhagia and bleeding disorders in its Title or Abstract. Once the search has narrowed to relevant articles only, the two students will independently conduct a full-text assessment to reinforce the studies' eligibility. The quality of the articles will also be analysed using various facets, such as the risk of bias. Nearer the end, the data will be synthesised in an orderly manner, making it easy for the reader to understand. The students will discuss the results to draw a conclusion from the systematic literature review data.

Most of the decisions will be made by the two medical students. If there are disagreements in deciding any aspect of the project, the supervisors will intervene to assist accordingly.

**Data extraction:**

Two reviewers (VD and RM) will work independently to collect data from each study from multiple electronic databases, transporting the data to a Microsoft Excel spreadsheet. A data extraction form will be piloted for standardisation purposes. The form will include information such as a unique identifier for the study involved, publication information (title, authors, journal, DOI, date, location), study type. Regarding any missing data, corresponding authors will be contacted to request access to the original raw data.

To consider the risk of bias (quality) assessment a quality assessment of each individual study will be conducted using the CASP checklist. The modified Newcastle-Ottawa Scale will be the formal risk of bias assessment tool used as our included studies will be non-randomised and mostly retrospective. Ideally, both reviewers will be involved in the quality assessment and any disputes managed by the supervisor.

**Data Synthesis**

The minimum number of studies for synthesis will be 2. The χ² test will be used to determine whether heterogeneity is present and possibly the I² test to measure the extent to which it exists. If meta-analysis is not possible due to a lack of available data or high heterogeneity, we may synthesise our data using the Synthesis Without Meta-analysis (SWiM) guidelines instead of a traditional narrative synthesis. This will allow us to combine our data using a standardised checklist, allowing increased consistency and transparency. The results may be grouped into types of bleeding disorders, management regimens used, or type of study eg qualitative or quantitative, depending on the articles retrieved.

**References:**

[Microsoft Word - Supplementary v3.docx (bmj.com)](https://bmjopen.bmj.com/content/bmjopen/suppl/2022/04/11/bmjopen-2021-057885.DC1/bmjopen-2021-057885supp001_data_supplement.pdf)
