# Supplementary material for: How should abnormal uterine bleeding be managed in people with bleeding disorders: a systematic review of the literature and thematic synthesis
Source: Res Pract Thromb Haemost. 2025 Sep 1;9(6):103167. doi: 10.1016/j.rpth.2025.103167 (PMC12495142; doi:10.1016/j.rpth.2025.103167)
Supplement: Supplemental Material 3 [file mmc4.docx]

**Reverse Snowballing**

| **Review article Author & Year** | **Article Title (+ link)** | **References included from articles** | **Points from articles** |
| --- | --- | --- | --- |
| Abdul-Kadir & Gomez, 2022 | Reproductive health and hemostatic issues in women and girls with congenital factor VII deficiency: A systematic review  <https://www.jthjournal.org/article/S1538-7836(22)18361-3/fulltext#secst0045> | Adeyemi‐Fowode O.A.Santos X.M.Dietrich J.E.Srivaths L. Levonorgestrel‐releasing intrauterine device use in female adolescents with heavy menstrual bleeding and bleeding disorders: single institution review. J Pediatr Adolesc Gynecol. 2017; 30: 479-483  Altay M.M., Haberal A.  Abnormal uterine bleeding in adolescents: Treatment with combined oral contraceptive pills is effective even in hospitalized patients with bleeding disorders. Turkish J Med Sci. 2008; 38: 431-435 | Long-acting contraceptives like the LNgIUD are better for managing HMB than daily hormonal therapy.  Oral contraceptives work well in patients with bleeding disorders |
| Ahuja & Hertweck, 2010 | Overview of Bleeding Disorders in Adolescent Females with Menorrhagia <https://doi.org/10.1016/j.jpag.2010.08.006> | Kingman C.E., Kadir R.A., Lee C.A. et al. The use of the levonorgestrel-releasing intrauterine system for treatment of menorrhagia in women with inherited bleeding disorders. BJOG. 2004; 111: 1425  Kouides P.A., Byams V.R., Phillip C.S. et al. Multisite management study of menorrhagia with abnormal laboratory haemostasis: a prospective crossover study of intranasal desmopressin and oral tranexamic. Br J Haematol. 2009; 145: 212 | LNG-IUS reduces the impact of menorrhagia  Neither treatment fully controlled menorrhagia |
| Borzutzky & Jaffray, 2020 | Diagnosis and Management of Heavy Menstrual Bleeding and Bleeding Disorders in Adolescents  <https://jamanetwork.com/journals/jamapediatrics/fullarticle/2757556> | Non-relevant, did not meet inclusion criteria, or have already been included elsewhere | Non-relevant, did not meet inclusion criteria, or have already been included elsewhere |
| Brignardello-Petersen et al, 2022 | Gynecologic and obstetric management of women with von Willebrand disease: summary of 3 systematic reviews of the literature  <https://ashpublications.org/bloodadvances/article/6/1/228/477423/Gynecologic-and-obstetric-management-of-women-with> | Non-relevant, did not meet inclusion criteria, or have already been included elsewhere | Non-relevant, did not meet inclusion criteria, or have already been included elsewhere |
| Curry et al, 2022 | Gynaecological management of women with inherited bleeding disorders. A UK Haemophilia Centres Doctors' Organisation Guideline  <https://onlinelibrary.wiley.com/doi/full/10.1111/hae.14643?saml_referrer> | Pennesi CM, Quint EH, Rosen MW, Compton SD, Odukoya EJ, Weyand AC. Outpatient Management of Heavy Menstrual Bleeding in Adolescent and Young Women with Inherited Platelet Function Disorders. J Pediatr Adolesc Gynecol. 2020; 33:489e493490.  Zia A, Kouides P, Khodyakov D, et al. Standardizing care to manage bleeding disorders in adolescents with heavy menses—A joint project from the ISTH pediatric/neonatal and women's health SSCs. J Thromb Haemostas. 2020; 18: 2759-2774.  Kadir RA, Lukes A, Kouides P, Fernandez H, Goudemand J. Management of excessive menstrual bleeding in women with haemostatic disorders. Fertility and Sterility. 2005; 84(5): 1352-1359. | No single best treatment was identified  Options should be individualized  Cyclical progestogens, ethamsylate, danazol and GnRH agonists can reduce menstrual blood loss but have different levels of effectiveness and side effects |
| Davies & Kadir, 2017 | Heavy menstrual bleeding: An update on management.  <https://www.sciencedirect.com/science/article/pii/S0049384817300725> | Non-relevant, did not meet inclusion criteria, or have already been included elsewhere | Non-relevant, did not meet inclusion criteria, or have already been included elsewhere |
| Deligeoroglou & Karountzos, 2018 | Abnormal Uterine Bleeding including coagulopathies and other menstrual disorders  <https://www.sciencedirect.com/science/article/pii/S1521693417301475?via%3Dihub> | Non-relevant, did not meet inclusion criteria, or have already been included elsewhere | Non-relevant, did not meet inclusion criteria, or have already been included elsewhere |
| Dickerson, Menon & Zia, 2018 | Abnormal Uterine Bleeding in Young Women with Blood Disorders  <https://www.sciencedirect.com/science/article/pii/S0031395518300300?via%3Dihub> | Non-relevant, did not meet inclusion criteria, or have already been included elsewhere | Non-relevant, did not meet inclusion criteria, or have already been included elsewhere |
| Djambas Khayat et al, 2020 | Heavy menstrual bleeding in women with inherited bleeding disorders  <https://onlinelibrary.wiley.com/doi/10.1111/hae.13888> | Non-relevant, did not meet inclusion criteria, or have already been included elsewhere | Non-relevant, did not meet inclusion criteria, or have already been included elsewhere |
| Eising, 2023 | Prophylactic and therapeutic strategies for intraoperative bleeding in women with von Willebrand disease and heavy menstrual bleeding: A systematic review  <https://www.sciencedirect.com/science/article/pii/S0268960X23000929> | H.P. Eising, Y.V. Sanders, J. de Meris, F.W.G. Leebeek, K. Meijer ‘Women prefer proactive support from providers for treatment of heavy menstrual bleeding: a qualitative study in adult women with moderate or severe Von Willebrand disease’ Haemophilia, 24 (6) (2018), pp. 950-956, [10.1111/hae.13552](https://doi.org/10.1111/hae.13552)  From NLM Medline | It is crucial to involve patients in decision-making for their HMB |
| Halimeh, 2015 | Menorrhagia and postpartum haemorrhage in women with rare bleeding disorder.  <https://www.sciencedirect.com/science/article/pii/S0049384815504386> | J.L. Kujovich  von Willebrand's disease and menorrhagia: prevalence, diagnosis, and management  Am J Hematol, 79 (2005), pp. 220-228 | Oral contraceptives, antifibrinolytics and desmopressin can be of assistance in the management of menorrhagia |
| Hayward, 2006 | Congenital platelet disorders: Overview of their mechanisms, diagnostic evaluation and treatment  <https://onlinelibrary.wiley.com/doi/10.1111/j.1365-2516.2006.01270.x> | Non-relevant, did not meet inclusion criteria, or have already been included elsewhere | Non-relevant, did not meet inclusion criteria, or have already been included elsewhere |
| Hermans et al, 2024 | Women and girls with inherited bleeding disorders: Focus on haemophilia carriers and heavy menstrual bleeding. Hermans C, Johnsen JM, Curry N. Haemophilia. 30 Suppl 3:45-51, 2024 Apr. PMID 38532560  <https://onlinelibrary.wiley.com/doi/10.1111/hae.14983> | Kuthiala S, Grabell J, Relke N, et al. Management of heavy menstrual bleeding in women with bleeding disorders in a tertiary care center. Res Pract Thromb Haemost. 2023; 7(3):100119. | There is a growing need for more effective treatment options and guidelines for managing HMB |
| James & Goodeve, 2011 | Von Willebrand disease  <https://www.gimjournal.org/article/S1098-3600(21)04779-1/fulltext> | Non-relevant, did not meet inclusion criteria, or have already been included elsewhere | Non-relevant, did not meet inclusion criteria, or have already been included elsewhere |
| Kadir & Chi, 2007 | Levonorgestrel intrauterine system: bleeding disorders and anticoagulant therapy  <https://www.sciencedirect.com/science/article/pii/S0010782407000649> | Z.E. Schaedel, G. Dolan, M. Powell  The use of the levonorgestrel-releasing intrauterine system in the management of menorrhagia in women with hemostatic disorders  Am J Obstet Gynecol, 193 (2005), pp. 1361-1363 | LNG-IUS is good for managing menorrhagia in patients with haemostatic disorders |
| Kadir et al, 2012 | Inherited bleeding disorders in older women  <https://www.sciencedirect.com/science/article/pii/S0378512212000953> | Non-relevant, did not meet inclusion criteria, or have already been included elsewhere | Non-relevant, did not meet inclusion criteria, or have already been included elsewhere |
| Kontogiannis, A. et al., 2023 | Primary Hemostasis Disorders as a Cause of Heavy Menstrual Bleeding in Women of Reproductive Age | Huguelet P.S., Laurin J., Thornhill D., Moyer G. Use of the Levonorgestrel Intrauterine System to Treat Heavy Menstrual Bleeding in Adolescents and Young Adults with Inherited Bleeding Disorders and Ehlers-Danlos Syndrome. J. Pediatr. Adolesc. Gynecol. 2021;35:147–152.e1. doi: 10.1016/j.jpag.2021.11.002. | High long-term continuation rate for LNG-IUS |
| Kouides, P.A., 2003 | Females with von Willebrand disease: 72 years as the silent majority  <https://onlinelibrary.wiley.com/doi/epdf/10.1046/j.1365-2516.1998.440665.x> | Non-relevant, did not meet inclusion criteria, or have already been included elsewhere | Non-relevant, did not meet inclusion criteria, or have already been included elsewhere |
| Kouides, P.A., 2005 | Kouides, P.A.  Von Willebrand Disease and other Disorders of Hemostasis in the Patient with Menorrhagia  <https://journals.sagepub.com/doi/10.2217/17455057.1.2.231?url_ver=Z39.88-2003&rfr_id=ori:rid:crossref.org&rfr_dat=cr_pub%20%200pubmed> | Non-relevant, did not meet inclusion criteria, or have already been included elsewhere | Non-relevant, did not meet inclusion criteria, or have already been included elsewhere |
| Kouides, P.A., 2006 | Current understanding of von Willebrand's disease in women - Some answers, more questions  <https://onlinelibrary.wiley.com/doi/10.1111/j.1365-2516.2006.01272.x> | Stewart A, Cummins C, Gold L, Jordan R, Phillips W. The effectiveness of the levonorgestrel-releasing intrauterine system in menorrhagia: a systematic review. Br J Obstet Gynaecol 2001; 108: 74–86. | LNG-IUS can reduce surgery rates and costs |
| Kouides, P.A. and Kadir R.A., 2007 | Menorrhagia associated with laboratory abnormalities of hemostasis: Epidemiological, diagnostic and therapeutic aspects  <https://www.jthjournal.org/article/S1538-7836(22)17584-7/fulltext> | Non-relevant, did not meet inclusion criteria, or have already been included elsewhere | Non-relevant, did not meet inclusion criteria, or have already been included elsewhere |
| Kujovich, J.L., 2005 | von Willebrand's disease and menorrhagia: prevalence, diagnosis, and management  <https://onlinelibrary.wiley.com/doi/10.1002/ajh.20372> | Non-relevant, did not meet inclusion criteria, or have already been included elsewhere | Non-relevant, did not meet inclusion criteria, or have already been included elsewhere |
| Kulkarni, R., 2015 | Improving care and treatment options for women and girls with bleeding disorders.  <https://onlinelibrary.wiley.com/doi/10.1111/ejh.12580> | Kouides PA, Byams VR, Philipp CS, et al. Multisite management study of menorrhagia with abnormal laboratory haemostasis: a prospective crossover study of intranasal desmopressin and oral tranexamic acid. Br J Haematol 2009; 145: 212–20. | IN-DDAVP showed a non-significant improvement in menstrual scores |
| Laffan, M. Sathar, J. and Johnsen, J.M., 2021 | von Willebrand disease: Diagnosis and treatment, treatment of women, and genomic approach to diagnosis.  https://onlinelibrary.wiley.com/doi/10.1111/hae.14050 | Non-relevant, did not meet inclusion criteria, or have already been included elsewhere | Non-relevant, did not meet inclusion criteria, or have already been included elsewhere |
| Lee, C.A., 2002 | Women and von Willebrand disease  <https://onlinelibrary.wiley.com/doi/full/10.1046/j.1365-2516.1999.0050s2038.x> | Non-relevant, did not meet inclusion criteria, or have already been included elsewhere | Non-relevant, did not meet inclusion criteria, or have already been included elsewhere |
| Lee, C.A. et al., 2006 | The obstetric and gynaecological management of women with inherited bleeding disorders--review with guidelines produced by a taskforce of UK Haemophilia Centre Doctors' Organization  https://haemophilia.org.uk/wp-content/uploads/2018/06/j.1365-2516.2006.01314.x.pdf | Chi C, Shiltagh N, Kingman CEC, Economides DL, Lee CA, Kadir RA. Identification and mangement of women with inherited bleeding disorder: A survey of obstetrician and gynaecologists in the United Kingdom. Haemophilia 2006; 12: 405–412.  Kadir RA, Lukes AS, Kouides PA, Fernandez H, Goudemand J. Management of excessive menstrual bleeding in women with hemostatic disorders. Fertil Steril 2005; 84: 1352–1359. | Desmopressin (DDAVP) helps manage bleeding disorders  Combined oral contraceptives help reduce menstrual blood loss |
| Leebeek, F.W.G., 2023 | Heavy menstrual blood loss in patients with von Willebrand disease: an unsolved problem  https://www.sciencedirect.com/science/article/pii/S2352302623001655 | Non-relevant, did not meet inclusion criteria | Non-relevant, did not meet inclusion criteria |
| Leissinger, C. et al., 2014 | Desmopressin (DDAVP) in the management of patients with congenital bleeding disorders  <https://onlinelibrary.wiley.com/doi/10.1111/hae.12254> | Non-relevant, did not meet inclusion criteria | Non-relevant, did not meet inclusion criteria |
| Lethagen, S., 2001 | Desmopressin in the treatment of women's bleeding disorders  <https://onlinelibrary.wiley.com/doi/full/10.1046/j.1365-2516.1999.00313.x?sid=nlm%3Apubmed> | Non-relevant, did not meet inclusion criteria | Non-relevant, did not meet inclusion criteria |
| Matthews, D.C., 2013 | Inherited disorders of platelet function  <https://www.sciencedirect.com/science/article/pii/S0031395513001156?via%3Dihub> | S.S. Rose, A. Faiz, C.H. Miller, et al. Laboratory response to intranasal desmopressin in women with menorrhagia and platelet dysfunction. Haemophilia, 14 (3) (2008), pp. 571-578 | IN-DDAVP treatment led to increased VWF and FVIII levels |
| McLintock, C., 2018 | Women with bleeding disorders: Clinical and psychological issues.  https://onlinelibrary.wiley.com/doi/10.1111/hae.13501 | Non-relevant, did not meet inclusion criteria | Non-relevant, did not meet inclusion criteria |
| Mikhail, S. and Kouides, P., 2010 | Von Willebrand Disease in the Pediatric and Adolescent Population. <https://www.sciencedirect.com/science/article/pii/S1083318810002494> | Non-relevant, did not meet inclusion criteria | Non-relevant, did not meet inclusion criteria |
| O'Flynn O'Brien, K.L., 2021 | Provider Attitudes and Practices Regarding Intrauterine System (IUS) Insertion in Adolescents With and Without Bleeding Disorders for Management of Heavy Menstrual Bleeding  <https://www.jpagonline.org/article/S1083-3188(21)00016-4/abstract> | Kingman C.E. Kadir R.A. Lee C.A. et al. The use of levonorgestrel-releasing intrauterine system for treatment of menorrhagia in women with inherited bleeding disorders. BJOG. 2004; 111: 1425 | Intranasal DDAVP can help increase factor VIII and von Willebrand factor |
| Oliveira et al, 2024 | Heavy menstrual bleeding in women with inherited bleeding disorders in use of LNG-IUS: A systematic review and single-arm meta-analysis  <https://www.contraceptionjournal.org/article/S0010-7824(24)00113-6/abstract#secsect0115> | Non-relevant, did not meet inclusion criteria, or have already been included elsewhere | Non-relevant, did not meet inclusion criteria, or have already been included elsewhere |
| Paper, R., 2000 | Gynaecological complications in women with bleeding disorders  <https://onlinelibrary.wiley.com/doi/full/10.1046/j.1365-2516.2000.00043.x?sid=nlm%3Apubmed> | Non-relevant, did not meet inclusion criteria | Non-relevant, did not meet inclusion criteria |
| Pennesi, C.M. et al., 2020 | Outpatient Management of Heavy Menstrual Bleeding in Adolescent and Young Women with Inherited Platelet Function Disorders  <https://www.jpagonline.org/article/S1083-3188(20)30256-4/abstract#secsectitle0070> | Dowlut-McElroy T. Williams K.B. Carpenter S.L. et al. Menstrual patterns and treatment of heavy menstrual bleeding in adolescents with bleeding disorders. J Pediatr Adolesc Gynecol. 2015; 28: 499 | Many prepubertal girls with bleeding disorders do not have a plan for managing HMB |
| Philipp, C.S., 2010 | Platelet Disorders in Adolescents  <https://www.sciencedirect.com/science/article/pii/S1083318810002561> | Non-relevant, did not meet inclusion criteria | Non-relevant, did not meet inclusion criteria |
| Philipp, C.S., 2011 | Antifibrinolytics in women with menorrhagia  https://www.sciencedirect.com/science/article/pii/S0049384811700305 | Non-relevant, did not meet inclusion criteria | Non-relevant, did not meet inclusion criteria |
| Presky, K.O. and Kadir, R.A., 2019 | Women with inherited bleeding disorders – Challenges and strategies for improved care  <https://www.thrombosisresearch.com/article/S0049-3848(19)30287-7/abstract> | James A.H., Kouides P.A., Abdul-Kadir R. et al. Evaluation and management of acute menorrhagia in women with and without underlying bleeding disorders: consensus from an international expert panel. Eur. J. Obstet. Gynecol. Reprod. Biol. 2011; 158: 124-134  Adeyemi-Fowode O.A. Santos X.M. Dietrich J.E. et al. Levonorgestrel-releasing intrauterine device use in female adolescents with heavy menstrual bleeding and bleeding disorders: single institution review. J. Pediatr. Adolesc. Gynecol. 2017 Aug; 30 (Epub 2016 Apr 21): 479-483 https://doi.org/10.1016/j.jpag.2016.04.001 | Anticoagulation should be restarted carefully, where antifibrinolytics are contraindicated  All patients experienced significant improvement in menorrhagia after LNgIUD placement |
| Ray, S. and Ray, A., 2016 | Non-surgical interventions for treating heavy menstrual bleeding (menorrhagia) in women with bleeding disorders  <https://www.ncbi.nlm.nih.gov/pmc/articles/PMC6734121/> | Kouides PA, Byams RV, Philipp CS, Stein SF, Heit JA, et al. Multisite management study of menorrhagia with abnormal laboratory haemostasis: a prospective crossover study of intranasal desmopressin and oral tranexamic acid. British Journal of Haematology 2009;145(2):212‐20. | Combining IN-DDAVP and TA with medical interventions could improve treatment outcomes for menorrhagia. |
| Rodeghiero, F., 2008 | Management of menorrhagia in women with inherited bleeding disorders: general principles and use of desmopressin. | Shankar M, Lee CA, Sabin CA, Economides DL, Kadir RA. von Willebrand disease in women with menorrhagia: a systematic review. BJOG 2004; 111: 734–40. | Increased awareness and collaboration between gynaecologists and haematologists are vital |
| Siegel, J.E. and Kouides, P.A., 2002 | Menorrhagia from a haematologist's point of view. Part II: management  <https://onlinelibrary.wiley.com/doi/full/10.1046/j.1365-2516.2002.00621.x?sid=nlm%3Apubmed> | Kadir RA, Economides DL, Lee CA. DDAVP nasal spray for treatment of menorrhagia in women with inherited bleeding disorders: a prospective randomized placebo-controlled cross-over study. Haemophilia 2000; 6: 243243. | Combining DDAVP nasal spray with antifibrinolytics might be a promising approach |
| Winikoff, R. et al., 2004 | The role of haemophilia treatment centres in providing services to women with bleeding disorders  https://onlinelibrary.wiley.com/doi/full/10.1111/j.1365-2516.2004.01001.x?sid=nlm%3Apubmed | Kouides PA. Females with von Willebrand disease: 72 years as the silent majority. Haemophilia 1998; 4: 665–76.DOI: 10.1046/j.1365-2516.1998.440665.x  Ragni MV, Bontempo FA, Hassett AC. von Willebrand disease and bleeding in women. Haemophilia 1999; 5: 313–7.DOI: 10.1046/j.1365-2516.1999.00342.x  Kingman CEC et al. The use of Levonorgestrel-releasing intrauterine system for the treatment of menorrhagia in women with inherited bleeding disorders. Br J Obstet Gynaecol 2004; in press. | Women with a history of menorrhagia experienced postoperative bleeding after procedures  Among 12 women who bled after non-gynecological procedures, a vWD diagnosis was known in only one  Tranexamic acid is effective but requires high doses |
