# Supplementary material for: How should abnormal uterine bleeding be managed in people with bleeding disorders: a systematic review of the literature and thematic synthesis
Source: Res Pract Thromb Haemost. 2025 Sep 1;9(6):103167. doi: 10.1016/j.rpth.2025.103167 (PMC12495142; doi:10.1016/j.rpth.2025.103167)
Supplement: Supplemental Material 6 [file mmc7.pdf]

1. **Use of a levonorgestrel 52-mg intrauterine system in the control of abnormal uterine bleeding in women with inherited bleeding disorders**

Theme 1 - 'The use of LNG 52-mg IUS was effective in reducing menstrual bleeding in the women with inherited bleeding disorders by 3- months from baseline.'

'At 3 months after insertion, 9/21 women (43%) reported amenorrhea. At 6 and 12months, 15/20 women (75%) and 14/20 women (70%) reported amenorrhea, respectively.'

'The results from our study suggest that women with inherited bleeding disorders could benefit from the use of LNG 52-mg IUS for bleeding control, similar to women without bleeding disorders.'

Theme 2 - 'Women with inherited bleeding disorders are generally administered a treatment with combined oral contraceptives, tranexamic acid, and desmopressin (DDAVP) (in cases of von Willebrand disease and hemophilia A). All of these, isolated or combined, have shown some effectiveness in controlling heavy menstrual bleeding. Nonetheless, the success depends on the womens adherence to the treatment, and there might be some systemic side effects.'

Theme 3 - 'There was an improvement in all eight parameters of quality of life ($p<0.001$). The mean hemoglobin, ferritin, and serum iron levels were also higher at 12months than before LNG 52-mg IUS placement.'

'the increase in the scores in all eight health dimensions of the SF-36 demonstrated improvements in the womens quality of life as a result of adequate control of heavy menstrual bleeding.'

Another link with QoL could be considering iron levels: 'our study showed that the use of LNG 52-mg IUS was also effective in increasing the hemoglobin, ferritin, and serum iron levels in the women with inherited bleeding disorders after a minimum of 12months of use. This result is particularly important considering that these women are at a high risk of presenting anemia.'

~~Theme 4~~

2. **Levonorgestrel-releasing intrauterine system for the management of heavy menstrual bleeding in women with inherited bleeding disorders: long-term follow-up**

Theme 1 - 'Our study shows that the LNG-IUS provides an effective long-term treatment for HMB, as assessed by using the PBAC, in women with IBDs. All women had a PBAC score less than 100, and 42% of the women became amenorrheic with the LNG-IUS. This study also shows that the LNG-IUS is useful in the treatment of dysmenorrhea. Fourteen women had moderate to severe dysmenorrhea prior to treatment compared to only one after treatment.'

'It can be easily inserted in a clinic setting and is well accepted by the women. It should be offered to women with IBDs who also require contraception and certainly prior to surgical options.'

Theme 2 - 'Prior to receiving the LNG-IUS, 85% (22/ 26) of women had tried other treatment modalities for HMB, including tranexamic acid, the combined oral contraceptive pill, oral progesterone and desmopressin, which they did not find effective, and 62% (16/26) were on a combination of two or more of these treatments.'

Theme 3 - 'The scores for all categories included in the QOL questionnaire were significantly improved following treatment ($p\leq.01$). Prior to LNG-IUS insertion, 44% (8/18) rated their general health as poor

compared to none at follow-up. Both the total health and activity scores A and B (Table 2) improved with the LNG-IUS. Severe or very severe pain during menstruation was reported by 39% (7/18) of women before LNG-IUS use compared to none at follow-up. The total QOL scores increased from a median of 26 (range, 13– 48) to 52 (range, 39–59) with the LNG-IUS.'

~~Theme 4 -~~

## 3. Gynaecological and obstetric bleeding in moderate and severe von Willebrand disease

Theme 1 – mentioned

Theme 2 – discusses about other treatments used such as hysterectomy, endometrial ablation, desmopression. (mentioned)

Theme 3 – mentioned (Tosetto Bleeding Score)

Theme 4 - 'Despite the increased awareness of bleeding problems in women with VWD in the last decades, PPH is still a major concern in these women.'

'Remarkably, improvement of care and guidelines has not decreased the frequency of PPH. The cause of PPH in women who received prophy- lactic treatment was unknown. Prospective studies are needed in order to improve outcome and to optimise current treatment guidelines.'

[talk about postpartum haemorrhage in our theme write-up and link it with the next stage of a woman's life?]

Maybe a point for the recommendations? 'A hysterectomy was complicated by bleeding more often if VWD had not yet been diagnosed. It is therefore of utmost importance that gynaecologists consider inherited bleeding abnormalities including VWD because in these women other treatment options, i.e. intranasal desmopressin and/ or tranexamic acid, might have resulted in less menstrual blood loss. In case surgery is still needed, desmopressin or FVIII/VWF concentrate can be given perioperatively to prevent bleeding com- plications.'

## 4. Assessment of an Electronic Intervention in Young Women with Heavy Menstrual Bleeding

~~Theme 1~~

~~Theme 2~~

~~Theme 3~~

Theme 4 - [could perhaps link this point with increasing awareness and trying new strategies for menorrhagia]: 'ITD is an excellent tool for adolescents with HMB and BD to allow self-monitoring, provider monitoring, and improve educational access through engaging technology; compliance with device use was associated with several parameters suggestive of improved clinical outcomes.'

'To our knowledge, this is the first study to specifically investigate use of an innovative technology in an adolescent population with HMB and BDs. Our study showed improved compliance and fewer hospital days related to use of the device during the time frame of study participation.'

'Adolescents who used the device (group 1) were much more likely to report fewer breakthrough bleeding episodes, miss fewer medications, and had fewer admissions related to their BD compared with those in group 2. This technology also allowed enrollees who consistently used the device to

easily access information on their ITD regarding the clinic, staff support, and provider support, when medical questions arose.'

'This ITD tool might serve as a model to other centers managing adolescents with HMB and BDs by virtue of its inherent adaptability, minimal need for training, low cost, and potential to improve patient compliance with treatment regimens. Future directions include assessing cost savings as a result of device utilization and improving device use with ongoing reminders from clinic staff.'

5. **Menstrual Patterns and Treatment of Heavy Menstrual Bleeding in Adolescents with Bleeding Disorders**

~~Theme 1 – mentions it briefly but not explicitly~~
Theme 2 - 'as the majority of girls who failed initial therapy in our cohort had been prescribed a single treatment modality, we recommend that strong consideration be given to combination non-hormonal and hormonal modalities for the treatment of HMB in girls with bleeding disorders.'

~~Theme 3~~
Theme 4 - 'consultation with a pediatric gynecologist and hematologist prior to menarche would be helpful for girls with known bleeding disorders to outline abnormal patterns of bleeding and to discuss options for treatment in the event of HMB at or after menarche. We propose that the creation of a national registry that standardizes the collection of menstrual data and treatment plans would further delineate menstrual patterns and effective treatment of HMB in girls with bleeding disorders. The data collected could then be used to develop guidelines for the optimal management of HMB for these girls.'

'Only 28% of the cohort had notations indicating that a treatment plan for heavy menstrual bleeding had been discussed with their provider prior to menarche (P 5 .0001)' [emphasis on more education and planning needed]

6. **Heavy menstrual bleeding and health-associated quality of life in women with von Willebrand's disease**

Theme 1 – mentioned 'In addition, LNG-IUS should be used more as a cessation of menstruation, since it often improves the quality of life.'

~~Theme 2 – mentioned briefly but not explicitly~~

Theme 3 - 'HMB affects numerous aspects of health-associated quality of life, both physical and mental. It can cause iron-deficiency anaemia in severe cases, and there are psychological, social and employment consequences associated with HMB'

'little is known about how such women perceive their health-associated quality of life, and whether there may be a potential benefit in a closer cooperation between hematologists and gynecologists.'

'Approximately one third of women (31.8%) with VWD were confined to bed for over half a day during the menstrual period.'

[points to consider for our recommendations/ write-up: 'Barr *et al* (14) demonstrated that women with VWD participate less in post-secondary education than the general population, and suggested

that this may be due to iron deficiency resulting from HMB. Côté *et al* (15) reported that HMB is also associated with a marked work loss and has important economic implications for women.'

Theme 4 - 'preventing limitations in overall life activities and improving their health-associated quality of life thorough counseling on menstrual bleeding is important for women with VWD.'

7. **Octreotide for the treatment of abnormal uterine bleeding in women with bleeding and clotting disorders**

~~Theme 1~~

Theme 2 – NB: Octreotide is a synthetic analogue of somatostatin. "Octreotide was effective in reducing AUB in 9 (82%) subjects who had failed oral contraceptives, Nexplanon implants, intrauterine devices and/or tranexamic acid. One patient with von Willebrand disease failed to respond to von Willebrand factor concentrate but responded to octreotide. Two (18%) subjects discontinued therapy before AUB could be assessed."

~~Theme 3~~

~~Theme 4~~

8. **Use of the Levonorgestrel Intrauterine System to Treat Heavy Menstrual Bleeding in Adolescents and Young Adults with Inherited Bleeding Disorders and Ehlers-Danlos Syndrome**

*Theme1* - "The 52-mg LNG-IUS is an effective treatment option for adolescents and young adults with heavy menstrual bleeding and a bleeding diathesis, with high rates of amenorrhea." "Overall, the majority of patients (81.8%, n = 27/33) reported improvement in bleeding, with 60.6% (n = 20/33) reporting spotting or amenorrhea at 6 months."

~~Theme2~~

~~Theme3 – Mentions more research needed but doesn't actually comment on QOL: *"Future studies are needed to determine if earlier insertion of the LNG-IUS would result in similar rates of amenorrhea and if overall patient quality of life could be improved with earlier placement."*~~

~~Theme4~~

9. **The outcome of endometrial ablation in women with inherited bleeding disorders**

*Theme1*

*Theme2 and theme 3* - “The median Hb concentrations (10.5–13.1 g dL−1) and QOL scores (median, 17–54) improved significantly after endometrial ablation ($P < 0.01$). Endometrial ablation appears to be a safe and effective long-term treatment for HMB in women with IDBs. It significantly decreases menstrual blood loss and improves QOL.”

“The median PBAC score decreased significantly from 1208 (range = 136–3850) preoperatively to 0 (range = 0–338) Postoperatively ($P = 0.002$)”. *NB: This can only be used in women that do not wish to preserve fertility.*

*Theme3* - “The overall scores for all categories included in the QOL improved significantly after treatment ($P < 0.01$). Prior to ablation, 67% rated their general health as poor compared with 0% at follow-up. Both the general health and activity scores A and B (Table 3) improved significantly after ablation. Severe or very severe pain during menstruation was reported in 58% (7/12) of women preablation compared with none Postoperatively. The total QOL scores increased from a median of 17 (range = 10–27) to 54 (range = 52–56) post-ablation ($P < 0.0001$).”

*Theme4* - “close liaison with the Haemophilia team is essential to determine the risk of bleeding and the need for haemostatic prophylaxis.”

10. **DDAVP nasal spray for treatment of menorrhagia in women with inherited bleeding disorders: a randomized placebo-controlled crossover study**

Theme 1

*Theme 2* - “After adjusting for this differences, mean PBAC scores were slightly lower (mean difference 8; 95% confidence interval of −15.5 to 31.6) in women receiving DDAVP than when receiving placebo, although this difference was not statistically significant ($P = 0.51$)”

Theme 3 – " the quality of life of the patients was better and the preference was for the second treatment; this may be related to the improved PBAC score or may be because the second treatment was fresh in their minds and was therefore selected in preference

to the treatment further in the past. Even weight fluctuation during the menstrual period was less pronounced and fluid intake was reduced in period 2 compared with period 1. However, there was no treatment (or treatment sequence) effect on PBAC scores, quality of life, adverse effects, changes in weight or fluid intake."

Yet… "A sufficiently long run-in period for women to get used to the trial procedures is also recommended. Assessment of efficacy should be based on measurement of menstrual blood loss of at least three consecutive periods as well as assessing the effect on patients' satisfaction and quality of life."

~~Theme 4~~

11. **High-dose DDAVP intranasal spray (Stimate®) for the prevention and treatment of bleeding in patients with mild haemophilia A, mild or moderate type 1 von Willebrand disease and symptomatic carriers of haemophilia A**

~~Theme1~~

*Theme2* - "When used for the treatment of menorrhagia, the efficacy of high-dose DDAVP intranasal spray (1.5 mg mL–1) was rated as 'excellent' after 655 (92%) of 721 daily uses." "the results of this large-scale, long-term, open-label clinical trial indicate that the high-dose DDAVP intranasal spray (1.5 mg mL–1) is safe and effective for the home-based treatment of bleeding episodes and as prophylaxis against bleeding in a wide range of patients with mild haemophilia A, mild or moderate type 1 vWD, symptomatic carriers of haemophilia A and patients with platelet-based bleeding disorders"

~~Theme3~~

~~Theme4~~

12. **Outpatient Management of Heavy Menstrual Bleeding in Adolescent and Young Women with Inherited Platelet Function Disorders**

*Theme1 and theme 2* - "the most effective treatment for HMB in young women with IPFDs was not identified"… "it was ultimately difficult to determine an optimal treatment method"

*Theme3* - "young women with HMB miss more days of school and have increased disruption of hobbies and activities and decreased sports participation because of menses, compared with their peers with normal menses… These effects on quality of life make control of bleeding that much more important, as well as provider acknowledgement of these effects while working to achieve control"

*Theme4*- "these findings provide guidance to clinicians in counseling and will help manage expectations for patients who present with HMB in the setting of an IPFD. Appropriate counseling in these patients should include that it would not be unexpected for a patient to need more than one treatment before control of bleeding is achieved."

**13. Recombinant von Willebrand Factor and Tranexamic Acid for Heavy Menstrual Bleeding in VWD: Randomized Crossover Trial**

~~Theme1~~

*Theme2*- "rVWF is inferior to TA in reducing HMB in subjects with mild or moderate VWD, with neither treatment showed a clinically relevant effect."

"The PBAC was significantly lower during 2 cycles with TA than during 2 cycles with rVWF"

"While rVWF was not superior to TA, it did reduce PBAC blood loss and may provide another approach to HMB management in those for whom TA, hormones, or DDAVP are ineffective or poorly tolerated, and, as such, adds evidence to the ASH/ISTH/NHF/WFH international guidelines"

*Theme3* – mentioned. QoL survey's conducted

*Theme4* - "These findings support the importance of discussing treatment options for HMB with patients based on their preferences and lived experience."

Expand on recommendations and make it flow - Rameen
Expand on discussion - make it flow - Rameen
Add a couple more sentences to limitations & make it flow - Rameen
~~Thematic analysis - wording of the quotes - Millie~~
~~Shorten data extraction table - Millie~~
Covidence exported happened or not - Rameen
~~NICE guidelines & obs gynae guidelines - Millie~~
~~Key Messages section – Millie~~
Reference - Millie
